# Brain-behavior correlates of rhythmic timing and auditory-motor synchronization in children with developmental coordination disorder: an EEG study

**DOI:** 10.3389/fnhum.2025.1602580

**Published:** 2025-06-05

**Authors:** Marija Pranjić, Jason Leung, Ka Lun Tam, Helene Polatajko, Timothy Welsh, Tom Chau, Michael Thaut

**Affiliations:** ^1^Music and Health Research Collaboratory, Faculty of Music, University of Toronto, Toronto, ON, Canada; ^2^Bloorview Research Institute, Holland Bloorview Kids Rehabilitation Hospital, Toronto, ON, Canada; ^3^Department of Occupational Science and Occupational Therapy, Rehabilitation Sciences Institute, Faculty of Medicine, University of Toronto, Toronto, ON, Canada; ^4^Centre for Motor Control, Faculty of Kinesiology & Physical Education, University of Toronto, Toronto, ON, Canada; ^5^Institute of Biomedical Engineering, University of Toronto, Toronto, ON, Canada; ^6^Institute of Medical Science and Rehabilitation Research Institute, Faculty of Medicine, University of Toronto, Toronto, ON, Canada

**Keywords:** developmental coordination disorder, dyspraxia, EEG, auditory perception, auditory-motor synchronization, rhythmic timing, music education

## Abstract

Vulnerabilities in motor control and sensorimotor timing are hallmarks of developmental coordination disorder (DCD). Although the positive effects of rhythmic entrainment on motor performance have been demonstrated in adults with movement disorders, interactions between auditory and motor systems have not been well characterized in children with DCD. We employed neuropsychological tests, caregiver reports, adaptive psychophysical procedures, finger-tapping paradigms, and electroencephalography (EEG) recordings to determine whether children with DCD have auditory-perceptual difficulties, whether rhythmic auditory cues can improve their motor performance, and whether extensive musical training contributes to enhanced auditory-motor abilities. Thirty-four children aged 7–11 years participated, including children with DCD and typically developing children with and without musical training. As hypothesized, children with DCD exhibited difficulties in rhythmic timing in both auditory-perceptual and motor tasks, especially compared to typically developing children with musical training. Notably, rhythmic auditory stimuli significantly improved motor performance across groups, which was linked to increased beta power and reduced functional connectivity in the ipsilateral fronto-central network compared to unpaced movements. Moreover, children with musical training consistently outperformed their DCD and TD peers across tasks and showed greater interhemispheric connectivity during auditory-motor synchronization, suggesting that rhythmic skills can be enhanced through practice. Our findings provide compelling evidence for the use of individually tailored auditory/rhythm-based interventions in children with DCD and highlight the positive effects of music education on auditory-motor development. Given the strong association between rhythm perception and movement, future studies should continue to investigate the link between auditory and motor skills to identify the profiles of children who are most likely to benefit from such interventions.

## Introduction

Perceptual timing is essential for efficient perception-action coupling and adaptive interactions with our surroundings. Differences in perceptual timing have been reported in many movement disorders (Avanzino et al., 2016; Hove and Keller, 2015), including developmental coordination disorder (DCD), a common neurodevelopmental condition affecting motor planning, coordination, and learning (American Psychiatric Association, 2013). In DCD, vulnerabilities in sensorimotor timing are most evident in tasks requiring movement synchronization to an external visual or auditory cue (e.g., moving to the beat) (Blais et al., 2021; Pranjić et al., 2024; Chiel et al., 1998; Whitall et al., 2008). Researchers have endeavored to explain the existing timing differences using the internal modeling deficit hypothesis (Adams et al., 2014), which suggests that motor difficulties may be attributed to deficiencies in constructing predictive models of action (Miall and Wolpert, 1996; Shadmehr et al., 2010). More recently, it was proposed that auditory-perceptual deficits may also be a core feature of DCD (Trainor et al., 2018), given the critical role of perceptual timing in sensory-motor integration. Auditory-perceptual differences have also been observed across several neurodevelopmental conditions that frequently co-occur with DCD, highlighting their potential role as a transdiagnostic marker (Lense et al., 1835).

While the literature concerning auditory-motor coupling in DCD is limited, a recent review suggests that children with DCD display increased variability when coordinating movements both with and without external auditory cues compared to their typically developing peers (Pranjić et al., 2023). However, it is unclear whether children with DCD have auditory-perceptual vulnerabilities and whether rhythmic auditory cues positively or negatively affect their motor performance. For example, children with DCD may also struggle in tasks requiring perceptual judgment in the absence of motor movement (i.e., auditory-perceptual timing) and/or in tasks requiring the integration of auditory feedback to predict and correct the timing of motor responses (i.e., auditory-motor timing).

In contrast, the mechanisms underlying auditory-motor coupling have extensively been studied in adults (Repp and Su, 2013), showing that even passively listening to rhythmic auditory stimuli can elicit brain activity in regions associated with motor functions (Fujioka et al., 2012; Grahn and Brett, 2007). These interactions arise through rhythmic entrainment, a process that occurs when the frequency of exogenous rhythmic auditory cues (e.g., metronome clicks) determines the frequency of neuronal oscillations in the motor system (Molinari et al., 2003; Thaut et al., 2015). Notably, the notion that rhythmic auditory stimuli can entrain motor responses has been increasingly applied in motor rehabilitation. For instance, auditory/rhythm-based therapeutic interventions have been shown to improve the stability of spatiotemporal patterns in adults with movement difficulties by providing a predictive time reference to the motor system, thereby decreasing the processing load (Thaut et al., 2015; Braun Janzen et al., 2022; Thaut and Abiru, 2010).

This research has also led to the development of test batteries aimed at assessing individual timing profiles through perception and production tasks (Fujii and Schlaug, 2013). These tests have proven valuable in detecting timing-related differences in neurotypical adults and point to an inverse relationship between tapping variability and perceptual abilities. Nonetheless, it remains uncertain whether the same mechanisms apply to children, given that auditory-motor interactions develop gradually and are shaped by the maturity of underlying neural networks (Trainor and Marsh-Rollo, 2019).

Interesting insights into auditory-motor interactions can be gained from studies on music learning since auditory feedback plays a crucial role in correcting movement patterns during playing (Zatorre et al., 2007). For example, long-term musical training has been associated with improvements in sound pattern recognition and fine motor skills, as well as increases in neuroplasticity across the lifespan, indicating that auditory-motor skills can be strengthened through extensive practice (Zatorre et al., 2007; Chen et al., 2008). Furthermore, musical training in childhood has been associated with enhanced literacy skills (Gordon et al., 2015), executive function performance (Rodriguez-Gomez and Talero-Gutiérrez, 2022), pitch discrimination abilities (Moreno et al., 2009), and even academic performance in adulthood (Miendlarzewska and Trost, 2014). In contrast, rhythmic timing abilities are often negatively affected in neurological movement disorders. Therefore, it remains to be investigated whether auditory/rhythm-based interventions can improve auditory-perceptual and motor performance in children with motor coordination difficulties.

In the current study, we used a selection of tasks from the Battery for the Assessment of Auditory Sensorimotor and Timing Abilities (BAASTA) (Dalla Bella et al., 2017) to examine the auditory-perceptual and auditory-motor timing abilities of children with DCD and their typically developing peers with and without extensive musical training. Specifically, we tested auditory-perceptual timing using duration and rhythm discrimination tasks and compared paced to unpaced tapping performance (i.e., tapping with and without rhythmic auditory cues) at fast (2 Hz) and slow (1 Hz) tempi. Using these tasks, we assessed (1) whether or not children with DCD have auditory-perceptual difficulties; (2) whether or not the addition of rhythmic auditory cues improves motor performance in children; and (3) whether or not extensive musical training in childhood is associated with enhanced auditory-perceptual and auditory-motor skills. We hypothesized that children with DCD would exhibit significantly lower perceptual acuity and increased motor variability compared to typically developing peers with and without musical training. We also expected that all groups would display decreased variability during paced compared to unpaced tapping and that musical training would be associated with enhanced performance across all tasks.

In addition to behavioral measures, we employed electroencephalography (EEG) recordings to examine functional brain connectivity and neural power spectra underpinning finger-tapping performance across groups and conditions. EEG is a non-invasive neurophysiological method that provides temporally sensitive information about cortical brain activity in response to internal and external stimuli. Brain connectivity can be assessed using coherence analysis by measuring the degree of neural oscillatory synchronization within and across different brain regions. Since unpaced tapping requires greater reliance on internal timing mechanisms (Serrien, 2008; Stegemöller et al., 2018), we examined the activity in the fronto-central network and hypothesized that tapping without auditory cues would be associated with increased intrahemispheric coherence compared to paced movements. No *a priori* hypotheses were made for the analysis of neural power spectra.

## Materials and methods

### Participants

A total of thirty-four children aged 7–11 years participated in the study (18 females, 16 males). The sample included 11 children with developmental coordination disorder (DCD), 11 typically developing children (TD), and 12 typically developing children with extensive musical training (TDM). We defined extensive musical training as a minimum of 2 years of formal piano lessons with regular practice at the time of enrollment. We specifically focused on piano lessons because of their strong emphasis on sensorimotor skills and bilateral movement dexterity. Participant characteristics are detailed in Table 1. Children in the DCD group had been screened for motor difficulties at a local hospital before participating in this study. The diagnostic criteria for DCD were further assessed according to the following DSM-5 standards (3): (A) a score at or below the 15^th^ percentile on the Movement Assessment Battery for Children, 2^nd^ Edition (MABC-2) (Henderson et al., 2007); and (B) a parent report confirming the presence of motor coordination difficulties and their effect on the child’s activities of daily living (DCD-Q) (Wilson et al., 2009). The inclusion criteria for typically developing children in both groups (i.e., TD and TDM) included a score above the 30^th^ percentile on the MABC-2 and a parent report indicating the absence of motor coordination difficulties. All children completed neuropsychological testing and scored above 70 on the Kaufman Brief Intelligence Test (Kaufman and Kaufman, 2013). Right-handedness was assessed using the revised version of the Edinburgh Handedness Inventory (Oldfield, 1971; Williams, 2020). Caregivers reported on the child’s developmental and medical history, musical training (e.g., the duration of musical training and the amount of practice per week, if applicable), and possible symptoms of ADHD and dyslexia. Exclusion criteria were other co-occurring diagnoses and/or medical conditions affecting hearing or motor functioning (e.g., cerebral palsy, hemiplegia, or muscular dystrophy). Considering that DCD and ADHD co-occur in approximately 50% of cases, children with coexisting DCD and ADHD symptomatology were eligible to participate (*n* = 3). Participants were recruited through the hospital’s research registry, pediatric occupational therapy clinics, local elementary schools, music schools, flyers, and word-of-mouth. All participants provided written assent, and their legal guardians provided written informed consent, in accordance with the Declaration of Helsinki and its later amendments. The study was approved by the Research Ethics Board at the Holland Bloorview Kids Rehabilitation Hospital and the University of Toronto. Participants received monetary compensation after the experiment.

**Table 1:** Demographic and clinical characteristics of participants by group.

	DCD	TD	TDM	*p*-value
*N*	11	11	12	-
Age (years)	9.09 ± 1.45	8.91 ± 1.38	9.42 ± 1.68	0.718
Race				
Caucasian	45.5%	54.5%	25%	0.100
Asian/Pacific Islander	9.1%	18.2%	58.3%	
Multiracial	36.4%	27.3%	8.3%	
Prefer not to say	9.1%	0%	8.3%	
Income				
<$49,999	9.1%	0%	0%	0.161
$50,000–$99,999	18.2%	18.2%	8.3%	
$100,000–$149,000	18.2%	9.1%	8.3%	
$150,000 or more	36.4%	36.4%	63.3%	
Prefer not to say	18.2%	36.4%	20.1%	
Laterality Quotient (RH)	90.91%	100%	98.88%	0.154
KBIT-2 Composite IQ	109.91 ± 19.42	115.91 ± 9.62	119.92 ± 8.82	0.273
DST Forward	7.73 ± 1.79	8 ± 1.61	8.92 ± 1.67	0.224
DST Backward	10.86	17.86	23.25	0.010^*^
MABC-2 Percentile	6.55 ± 4.40	54.73 ± 16.56	59.17 ± 12.56	<0.001^*^
DCD-Q	29.91 ± 8.10	66.64 ± 6.36	68.58 ± 5.81	<0.001^*^
ADHD-Q (SNAP-IV)	25.91 ± 16.60	9.45 ± 5.59	7.17 ± 4.32	0.007^*^
Musical Training (months)	2.27 ± 3.47	7.09 ± 6.74	52 ± 20	<0.001^*^

DCD, developmental coordination disorder; TD, typically developing children; TDM, typically developing musicians; Laterality Quotient (RH), right-handedness measures by Edinburgh Handedness Inventory; KBIT-2, The Kaufman Brief Intelligence Test, 2^nd^ Edition; MABC-2, The Movement Assessment Battery for Children, 2^nd^ Edition; DST, The Digit Span Test; DCD-Q, The Developmental Coordination Disorder Parent Questionnaire; SNAP-IV, The Swanson, Nolan, and Pelham questionnaire for ADHD identification. Difference tests were one-way ANOVAs (reported as M ± SD) and a Kruskal-Wallis test (Mean Rank reported for the DST Backward) for continuous variables, and chi-square tests for categorical variables. The responses on the Dyslexia Evaluation Checklist are not included in the table as they did not yield numeric scores and were assessed qualitatively.

* Indicates a significant difference between groups; *p* < 0.05.

### Procedures

Participants and their caregivers attended two research sessions, each lasting between 60 and 80 min. The first visit involved neuropsychological and motor testing and caregiver questionnaires to ensure the differences in cognitive functioning did not affect the performance on the experimental tasks. The second visit included behavioral tasks and EEG recordings to assess auditory-perceptual and auditory-motor timing abilities across groups.

#### Behavioral testing

Auditory-perceptual and auditory-motor timing skills were assessed using a selection of tasks from the BAASTA (Dalla Bella et al., 2017), with minor adjustments. We used two perceptual tasks that required participants to judge if there were differences in duration and rhythm between auditory stimuli (i.e., duration and rhythm discrimination) and two sensorimotor tasks that involved finger-tapping movements with and without rhythmic auditory cues (i.e., paced and unpaced tapping). The participants were seated in a relaxed position in a sound-attenuated room, free from distractions. Auditory stimuli were delivered via speakers located approximately 60 cm in front of the participants. The loudness of the auditory stimuli was tested at a 70 dB sound pressure level and was adjusted to match each participant’s preferred intensity level. The auditory stimulus was a 1,000 Hz tone for all tasks. Finger-tapping data were acquired via a Micro Light Switch by AbleNet (connected to the g. Nautilus box) that provided minimal auditory and tactile feedback to the participant. The tasks were presented in a fixed order (duration and rhythm discrimination, unpaced and paced tapping) to avoid the entrainment effect following rhythmic tasks (i.e., rhythm discrimination and paced tapping). The auditory-perceptual and finger-tapping paradigms each lasted approximately 20 min.

#### EEG recordings and processing

EEG data were recorded during the finger-tapping tasks. The data were acquired using the wireless g. Nautilus Research headset (g. SCARABEO, g.tec medical engineering GmbH, Austria) and the g.tec HIamp amplifier, which allowed combined recordings of EEG and fNIRS signal. Only the EEG data are presented here. EEG signals were recorded at 250 Hz from 32 active electrodes placed according to the modified international 10–20 system. Per manufacturer guidelines, the ground electrode was located at AFz, the reference was placed on the right earlobe, and the electrode impedances were kept below 50 kΩ. A wireless cap was chosen to capture the child’s performance in a less constrained manner. The data were transferred via Bluetooth to a receiving computer running MATLAB (R2017a). The EEG data were preprocessed using the Harvard Automated Processing Pipeline for Electroencephalography (HAPPE), version 4 (Gabard-Durnam et al., 2018), which involved removing 60 Hz electrical line noise using the CleanLine method (Tim Mullen) and bandpass filtering the data between 0.5–80 Hz using a Hamming windowed sinc finite impulse response filter. After bad channels were rejected, ocular and muscle artifacts were removed using the wavelet thresholding ICA method (Gabard-Durnam et al., 2018). Bad channels were interpolated prior to average referencing. Participants were provided with a short training period to reduce artifacts caused by eye blinks and muscle activity and were given opportunities to stretch between tasks.

### Measures

#### Neuropsychological and motor assessments

Cognitive abilities were assessed with the Kaufman Brief Intelligence Test, 2^nd^ Edition (KBIT-2) (Kaufman and Kaufman, 2013) to exclude the possibility that poor motor and/or perceptual skills are due to cognitive differences. The KBIT-2 is a standardized measure used to estimate verbal and nonverbal intelligence in individuals aged 4 to 90. Before the analysis, raw scores were converted into standard scores. The Digit Span Test was used to assess immediate auditory attention (Digits Forward) and working memory (Digits Backward) and ensure that potential perceptual differences across groups cannot be attributed to working memory vulnerabilities (Hilbert et al., 2015). Participants were asked to repeat increasing spans of digits in the same (Digits Forward) and the reverse order they were presented (Digits Backward). Testing ended when the child provided two incorrect responses within a given span length. The overall score was the sum of trials correctly recalled. The scores were reported separately for the forward and backward span tasks. The MABC-2 (Swanson et al., 2012) was used to evaluate the participants’ motor skills and confirm the presence or absence of motor coordination difficulties. Motor performance was assessed across three domains, including (1) manual dexterity, (2) aiming and catching, and (3) balance. The total test scores and their standard score equivalents were converted into percentiles based on the child’s age. Scores at or below the 15^th^ percentile indicated the presence of movement difficulty, with lower scores denoting more significant impairment (Ip et al., 2021).

#### Caregiver questionnaires

All caregivers completed a set of questionnaires inquiring about the child’s possible DCD, ADHD, and dyslexia-related symptoms. This included the Developmental Coordination Disorder Questionnaire (DCD-Q) (Wilson et al., 2009), the Dyslexia Evaluation Checklist: Parent Form (DEC) (Proctor et al., 2017), and the Swanson, Nolan, and Pelham questionnaire (SNAP-IV) for ADHD identification (Swanson et al., 2012).

#### Auditory-perceptual tasks

Auditory-perceptual sensitivity was measured for duration discrimination (i.e., interval-based timing) and rhythm discrimination (i.e., beat-based timing) tasks. Perceptual thresholds for each task were estimated using an adaptive two-alternative forced-choice paradigm (Kingdom and Prins, 2010) wherein children were instructed to detect a change in duration or rhythm between two tones or sequences, respectively. As per Chang et al. (2021), we employed an adaptive 2-up-1-down staircase procedure where two successive correct responses increased task difficulty on the subsequent trial, and one incorrect response decreased the difficulty, converging at a 70.7% discrimination accuracy (Levitt, 1971). In both tasks, participants provided their responses verbally (“yes” indicated the situation when the child detected a difference), and the experimenter pressed the corresponding key on the computer.

The duration discrimination task assessed the participant’s ability to detect changes in tone durations in the absence of an underlying beat. Each trial consisted of two tones separated by 1,120 ms (tone frequency = 1 Hz). The standard tone was presented first at a fixed duration of 500 ms, followed by the target tone that changed adaptively according to the 2-up-1-down algorithm, ranging between 260 and 500 ms in 15 ms step sizes. The target tone in practice and probe trials had a fixed duration of 250 ms (easy discrimination level). Participants were instructed to judge whether the two tones had the same duration or if the second tone was different (i.e., shorter or faster).

In the rhythm discrimination task, we measured participants’ ability to detect a temporal irregularity in an isochronous sequence of tones. Participants listened to two 5-tone sequences separated by 1,120 ms (tone duration = 80 ms). The standard (isochronous) sequence was presented first with a constant inter-onset interval (IOI) of 500 ms, followed by the target sequence with a constant IOI of 500 ms, except for the last IOI, which was always shorter. The time shift between the last two tones in the target sequence changed adaptively depending on the participant’s response and ranged between 335 and 500 ms, with a step size of 15 ms. For practice and probe trials, the last IOI in the target sequence was fixed at 250 ms. Participants were instructed to judge whether the two rhythmic sequences were the same or if the second sequence had an offbeat tone (i.e., irregularity).

The tasks were preceded by five practice trials with feedback to ensure participants understood the instruction and could detect differences at an easy discrimination level (i.e., 50% change in the stimulus). Each task consisted of 43 trials, with five probe trials randomly placed within the experiment. The probe trials were set at the same difficulty level as the practice trials to control for possible attention lapses during the experiment. Performance was considered valid if participants responded correctly to at least 4 of the 5 probe trials. The probe trials were excluded from the analysis.

#### Finger-tapping tasks

**Figure 1:**
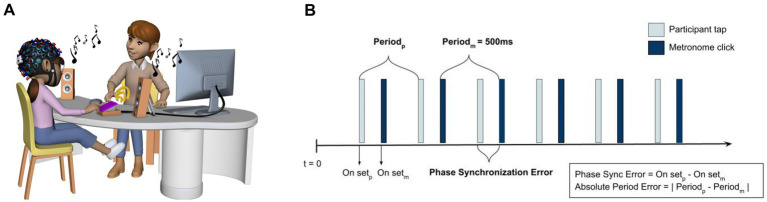
**(A)** Illustration of the finger tapping paradigms measuring motor and auditory-motor timing abilities. In the synchronization condition, participants were instructed to tap along with an auditory cue as accurately as possible. In the synchronization–continuation condition, participants were instructed to tap along with a sequence of 10 isochronous tones and then continue tapping at the same rate after the auditory cue stopped. Each tapping task was performed twice, yielding a total of eight tapping sequences (four synchronization sequences and four synchronization–continuation sequences). **(B)** Illustration of the phase synchronization error and absolute period error (adapted from Malcolm et al., 2008). Phase synchronization error refers to the difference between the onset of the metronome click and the finger tap; smaller values (in ms) indicate higher temporal accuracy. Absolute period error is the difference between the participant’s inter-tap interval and the fixed interval (500 ms) between metronome clicks.

Two finger-tapping tasks measured motor and auditory-motor timing abilities (Aschersleben, 2002; Repp, 2005) (see Figure 1). To assess the internal timing mechanisms, we employed the synchronization-continuation paradigm (i.e., unpaced tapping), where the participants were instructed to synchronize their tapping to a series of 10 isochronous tones and then continue tapping at the same rate after the auditory cue stops. The unpaced tapping was followed by the synchronization tasks (i.e., paced tapping) to assess the participant’s ability to temporally coordinate the movement to a predictable auditory cue (presented as metronome clicks). Both tasks were presented at a fast (IOI = 500 ms; 120 beats per minute) and a slow (IOI = 1,000 ms; 60 bpm) tempo. To ensure that the movements were simple to execute for all children, we chose in-phase tapping because it is considered more stable than anti-phase patterns. Participants were instructed to tap using their right hand.

Each task was repeated twice, with a total of eight tapping sequences (four synchronization sequences and four synchronization-continuation sequences). Before each tapping sequence, there was a 30-s resting period. The high-frequency beep sound (2,000 Hz) indicated the beginning and end of each tapping trial. The duration of each sequence corresponded to 60 IOIs/taps, wherein a slow condition lasted 60 s, and a fast condition was 30 s long. In both tapping tasks, the two sequences with the same tapping tempo were averaged to improve the stability of the values. The first 10 taps of each sequence were discarded from the analysis, leading to a total of approximately 100 taps in each condition. When the taps occurred less than 100 ms apart, the second tap was removed as it was considered an artifact (e.g., an accidental double tap (Dalla Bella et al., 2017). Additionally, any taps that were 50% longer or shorter than the target inter-tap intervals were excluded from the analysis (Koshimori et al., 2019).

### Data analysis

The perceptual thresholds for duration and rhythm discrimination tasks were calculated by averaging the values obtained across all trials for each task. The score was reported as the percentage of the stimulus IOI (Weber ratio), wherein the smaller number (ms) indicated better performance (i.e., higher perceptual acuity). In synchronization-continuation tapping tasks, only the continuation phase was analyzed. The mean inter-tap interval (ITI) was calculated as a measure of tapping variability (i.e., tapping rate), and its coefficient of variation (CV) reflected motor variability (calculated as the ratio of the SD of the ITIs over the mean ITI). For synchronization tasks, the mean phase synchronization error was computed as the absolute difference between the rhythmic auditory cue (metronome click) and the finger tap, reflecting how accurately the participants could align their taps with the auditory beat (Figure 1B). Synchronization variability was reported as the standard error (SE) (the SD of the synchronization error over the square root of the number of taps). Small synchronization errors (in ms) indicated high accuracy. To compare the performance characteristics of unpaced versus paced tapping, we calculated the absolute tapping period error and its standard deviation by extracting the difference between the absolute mean ITI and the auditory cue.

#### EEG data analysis

EEG analyses were performed using the MNE (MEG + EEG Analysis and Visualization) package with custom code written in Python. For frequency analysis, the EEG data were segmented into 350 ms epochs (−100 to 250 ms) around the tap onset for the fast condition (500 ms) and into 600 ms epochs (−100 to 500 ms) for the slow condition (1,000 ms). Baseline correction was applied by subtracting the average of the pre-movement onset between −300 to −100 ms for the fast condition and −500 to −300 ms for the slow condition. Since our study involved fast and slow tapping conditions, we modeled the baseline correction windows based on protocols from comparable paradigms (Stegemöller et al., 2021). All analyses were conducted in the beta band (12–30 Hz) due to its importance for sensorimotor behavior. The epochs for each condition were averaged together for each group before the power spectral density (PSD) was computed using the multitaper method (Gramfort et al., 2013). We computed the PSD from scalp locations overlying the precentral gyrus and/or supplementary motor area (Cz) and the primary motor cortex on the contralateral (C1, C5) and the ipsilateral side (C2, C6). Due to the placement of the fNIRS spacer pads, the C3/C4 electrodes were not included in the montage. For the plots, we first averaged the PSD across participants in each group and then applied a logarithmic transform of the PSD to dB for plotting. For connectivity analysis, the coherence between each channel pair, $C_{\mathrm{xy}}$ , was computed for each participant based on time-resolved estimates of cross-spectral densities ( $S_{\mathrm{xy}}$ ), and PSDs ( $S_{\mathrm{xx}}$ , $S_{\mathrm{yy}}$ ).

$$C_{\mathrm{xy}}=\frac{|E[S_{\mathrm{xy}}]|}{\sqrt{E[S_{\mathrm{xx}}]\times E[S_{\mathrm{yy}}]}}$$

We estimated the spectral connectivity over time as the coherence between selected channel pairs. The functional links resulted in the following groupings: intrahemispheric contralateral (Fp1-F3, F3-C5, C5-C1), intrahemispheric ipsilateral (Fp2-F4, F4-C6, C6-C2), and interhemispheric (Fp1-Fp2, F3-F4, C5-C6). The same time windows were used as earlier to epoch the data. The 30-s resting state EEG data were recorded before each tapping trial and were segmented into non-overlapping epochs of the same length as the task epochs. For each condition, the resting state coherence was subtracted from the tapping task coherence (Serrien and Brown, 2002). Coherence was estimated for the beta frequency band using Morlet wavelets with one cycle. Note that the channels PO7, PO3, PO4, PO8, and Oz were excluded from the analysis as the optode connector box was positioned in the back of the head, providing limited access to the occipital lobe. The difference plots (matrix style) were generated by computing the difference between the mean task and mean resting state coherence at each link. The two-sample Kolmogorov–Smirnov (KS) test was used to assess significant differences between the distribution of task and resting state coherence measures across epochs. The coherence differences were masked such that the coherence difference at a particular link was set to zero if the distribution of the task coherence was not statistically different from the resting state coherence based on the KS test. Note that the KS test was applied over epochs for each participant, and the *p*-value threshold was set to < 0.01. The resulting masked differences were then averaged across participants within the same group. Finally, the values of the selected links within each grouping were averaged before statistical analysis.

### Statistical analyses

Statistical analyses were computed using R Studio (version 4.3.0). The alpha level was fixed at *p* = 0.05 for all data analyses. Data normality was verified with the Shapiro–Wilk test, and the Kruskal-Wallis H was reported if the normality assumption was violated for at least two groups (*p* < 0.05). Levene’s Test was used to evaluate the homogeneity of variances assumption for each ANOVA. If significant (*p* < 0.05), we reported Welch’s test. Group differences were assessed using chi-square tests for categorical variables and one-way and mixed-design analyses of variance (ANOVAs) for continuous variables. The *lmer* function (*lme4* package in R) (Bates et al., 2015) was used to fit the ANOVA models. Additional analyses of covariance (ANCOVAs) were performed for perceptual and tapping data with age and working memory (Digit Span Backward) as covariates determined through bivariate correlations of the main study variables. When main or interaction effects were found, post-hoc paired and independent samples *t*-tests tests were computed using a Bonferroni correction. Two-tailed tests were reported for all analyses, except for the main effect of Task in the EEG analysis, given our *a priori* hypothesis regarding the direction of effects between paced and unpaced tapping.

## Results

### Participants

Of the 34 participants who completed the study, the tapping data from one participant in the DCD group were excluded as the participant did not follow the task instructions. We further excluded the EEG data from three participants in each group due to an unsatisfactory signal quality caused by excessive motion artifacts, as determined using the HAPPE Processing Report metrics (e.g., if the percentage of good channels was below 65% or if the electrodes overlying the primary cortex were heavily contaminated) (Gabard-Durnam et al., 2018). The groups did not significantly differ in the percentage of bad channels (*p* = 0.971) and the number of rejected epochs (all *p* > 0.05). Additionally, the number of excluded taps was comparable across groups, except in the slow unpaced condition (*p* = 0.002), where more taps were excluded in the DCD group (DCD 10.9 ± 5.8, TD 6.1 ± 3.7, TDM 4.1 ± 2.7). As detailed in Table 1, the three groups did not significantly differ in age, cognitive abilities, immediate attention (Digits Forward), race, and household income (all *p* > 0.05). For the Digits Backward Test, the data significantly deviated from normality for the DCD (*W* = 0.794, *p* = 0.008) and TDM groups (*W* = 0.852, *p* = 0.039). A Kruskal-Wallis test revealed significant group differences in working memory abilities [Digits Backward Test; *H*_(2)_ = 9.14, *p* = 0.010], with TD musicians scoring higher than children with DCD. However, this finding should be interpreted with caution as the group differences were only marginally significant after adjusting for age (*p* = 0.046). As expected, the MABC-2 confirmed the presence of motor difficulties for the DCD group only. Given that three participants with DCD had coexisting ADHD symptoms (caregiver report), we conducted additional analyses and found that participant characteristics and task performance did not significantly differ between the full DCD sample and the subgroup of children who had coexisting ADHD symptoms (DCD + ADHD). Children in the TD and TDM groups had no parent-reported ADHD or dyslexia symptoms.

### Auditory-perceptual timing

**Figure 2:**
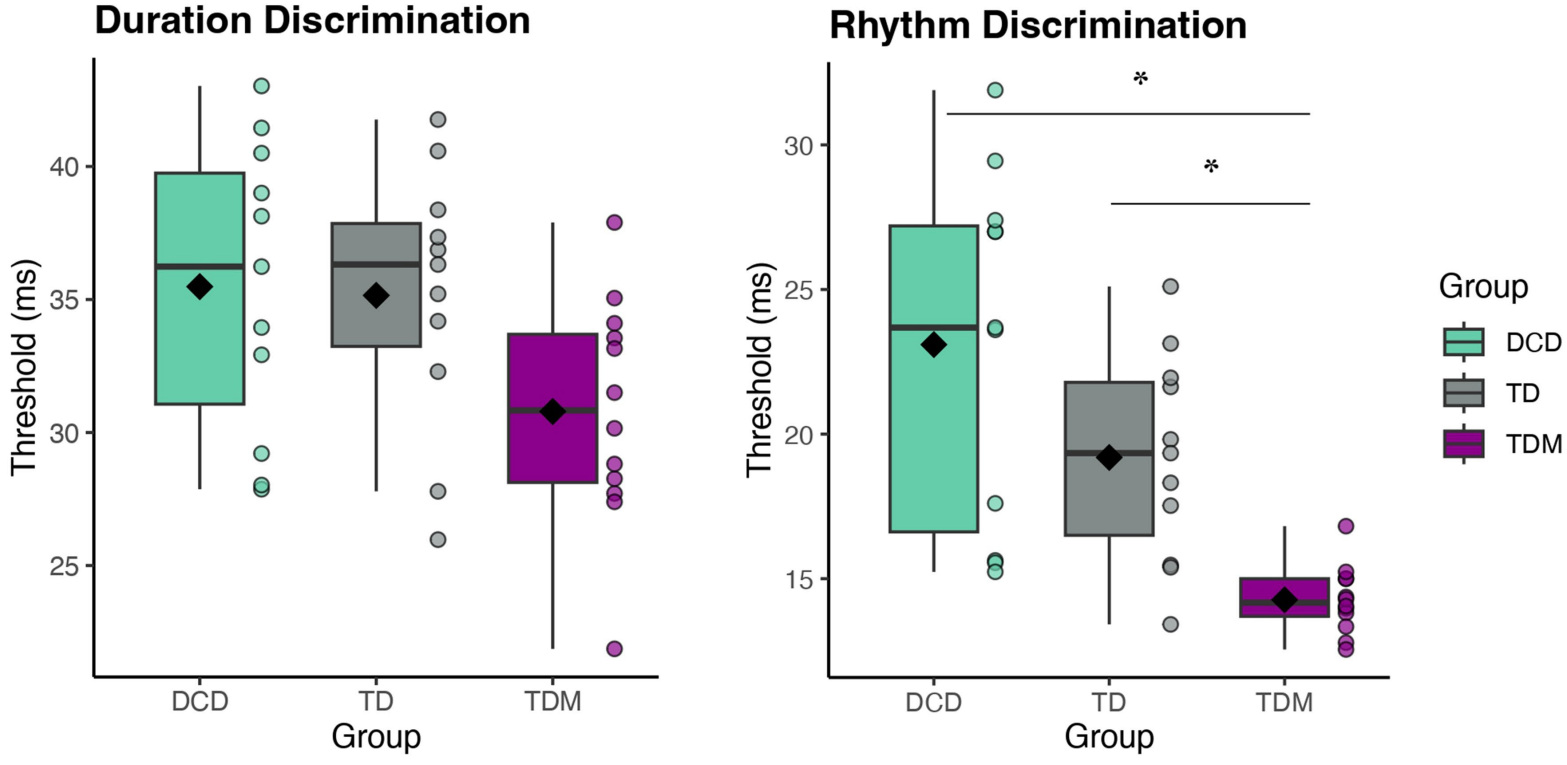
Distributions of perceptual thresholds for the duration (left) and rhythm discrimination (right) tasks by group. Participants were asked to discriminate differences in time durations between the pairs of tones (duration) and detect deviations from the beat in the rhythmic metronome sequence (rhythm). Significant group differences were found for the rhythm discrimination task, wherein children with TDM outperformed both children with DCD and their TD peers without musical training. *Note.* Lower thresholds indicate higher perceptual acuity. Scores are reported in milliseconds (ms) as a percentage of IOI% (Weber fraction). The box represents the interquartile range, the whiskers display minimum and maximum values (excluding outliers), the horizontal line represents the median, and the diamond shape indicates the mean value. Each dot represents one participant. *Denotes significance at *p*_adj_ < 0.016.

One-way ANOVAs revealed significant group differences for both perceptual tasks. For the duration discrimination thresholds [*F*_(2,31)_ = 3.33, *p* = 0.049, η_p_^2^ = 0.18], the musician group displayed smaller thresholds than both the DCD and TD groups, indicating higher perceptual acuity for interval-based timing. However, the group differences were no longer significant after the Bonferroni adjustment (see Figure 2). The assumption of homogeneity of variances was not met for the rhythm discrimination task [*F*_(2,31)_ = 10.56, *p* < 0.005], therefore the Welch’s test is reported. In the rhythm discrimination task [*F*_Welch(2,14.88)_ = 18.67, *p* < 0.001, η_p_^2^ = 0.47], musicians had significantly smaller perceptual thresholds than both children with DCD [*t*_(21)_ = 4.93, *p* < 0.001, *d* = 2.06] and their typically developing peers with no musical training [*t*_(21)_ = 4.50, *p* < 0.001, *d* = 1.88]. Although the thresholds for the TD and DCD groups did not significantly differ [*t*_(21)_ = 1.83, *p* = 0.082, *d* = 0.78], children with DCD had larger thresholds (23.10 ± 6.10) than the TD group (19.20 ± 3.60), indicating lower perceptual acuity. The group difference persisted after adjusting for age [*F*_(2,28)_ = 3.78, *p* = 0.035, η_p_^2^ = 0.21] (Figure 2).

### Finger-tapping performance

#### Unpaced tapping (motor timing)

When auditory cues were absent, children with DCD displayed significantly higher variability in maintaining the slow tapping rate compared to their TD peers [main effect of Group; *F*_(2,30)_ = 5.04, *p* = 0.013, η_p_^2^ = 0.25; DCD-TD; *t*_(19)_ = 4.50, *p* = 0.005, *d* = 1.38] and were more variable in the fast condition than TD and TDM children [main effect of Group; *F*_(2,30)_ = 9.99, *p* < 0.001, η_p_^2^ = 0.40; DCD-TD; *t*_(19)_ = 3.08, *p* = 0.006, *d* = 1.34; DCD-TDM; *t*_(20)_ = 3.96, *p* < 0.001, *d* = 1.70]. Interestingly, although TD and TDM groups displayed comparable tapping rates, TDMs were less variable during the slow condition (for details, see Supplementary Figure S1).

#### Paced tapping (auditory-motor timing)

Children with DCD were less accurate when synchronizing their taps to the auditory beat compared to TD and TDM groups in the fast condition [main effect of Group; *F*_(2,30)_ = 14.38, *p* < 0.001, η_p_^2^ = 0.49; DCD-TD; *t*_(19)_ = 3.24, *p* = 0.004, *d* = 1.42; DCD-TDM; *t*_(20)_ = 4.92, *p* < 0.001, *d* = 2.11], and compared to TD musicians in the slow condition [main effect of Group; *F*_Welch(2,30)_ = 7.34, *p* = 0.003, η_p_^2^ = 0.40; DCD-TDM; *t*_(20)_ = 3.65, *p* = 0.002, *d* = 1.56]. Children with musical training were also significantly more accurate in the slow condition than their TD peers without musical training (for details, see Supplementary Figure S2). Regarding tapping variability, children with musical training outperformed both groups in the fast and slow tapping conditions [main effect of Group; fast, *F*_(2,30)_ = 9.72, *p* < 0.001, η_p_^2^ = 0.39; slow, *F*_Welch(2,30)_ = 11.89, *p* = 0.001, η_p_^2^ = 0.44]. When covarying for age, the group differences persisted for the slow condition only.

#### Paced vs. unpaced tapping

**Figure 3:**
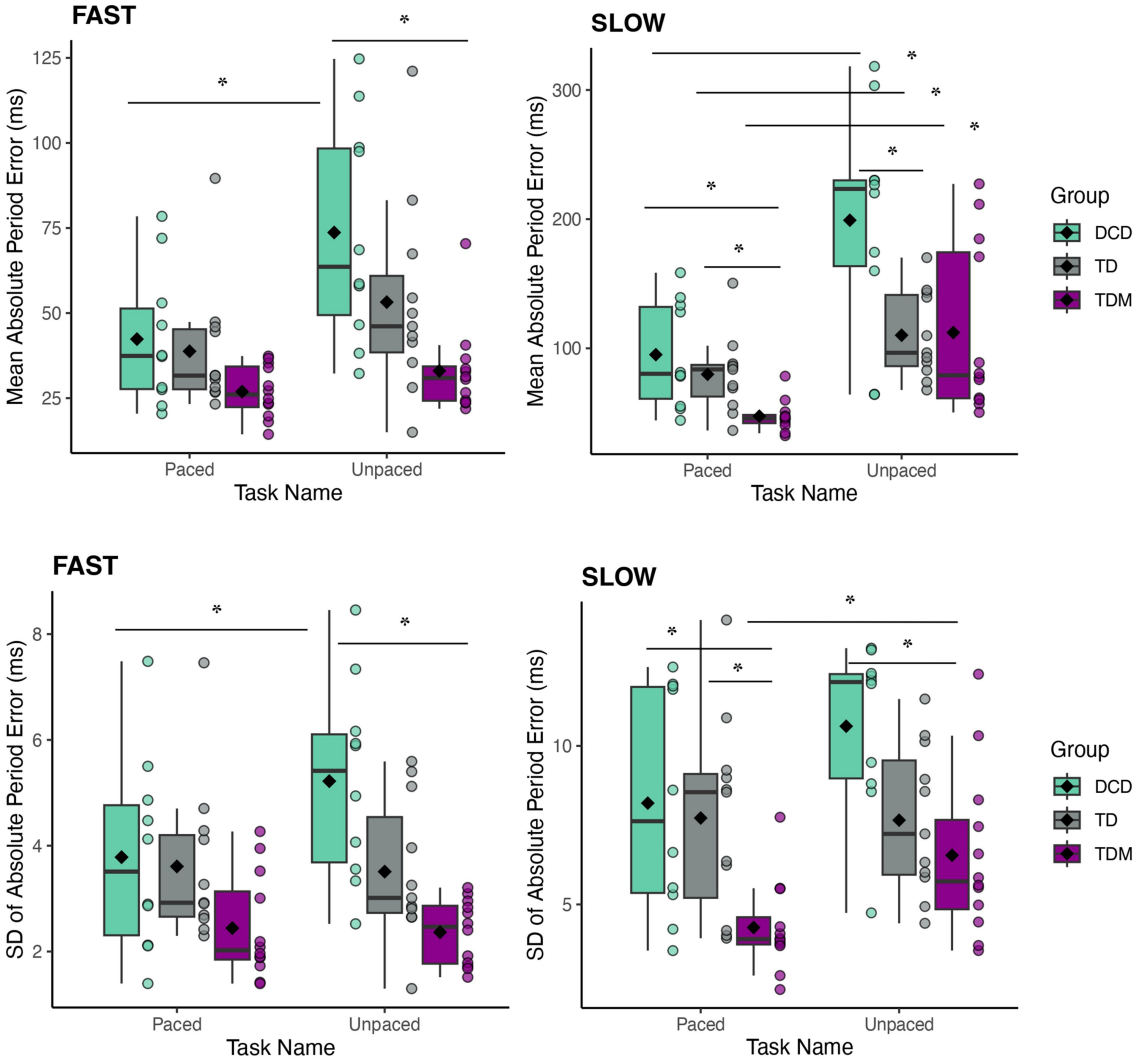
Comparisons between paced and unpaced tasks at fast (left) and slow (right) rates. Paced and unpaced tapping performance was compared to assess the effects of auditory stimuli on motor accuracy and variability. **(A)** Absolute tapping period error (APE) reflects the difference between the absolute mean ITI and the inter-stimulus interval. **(B)** Variability is reported as the standard deviation of APE. Lower values indicate less error **(A)** and variability **(B)**. The box represents the interquartile range, the whiskers display minimum and maximum values (excluding outliers), the horizontal line represents the median, and the diamond shape indicates the mean value. Each dot represents one participant. Note. While each dot represents a participant, please note that some individual data points overlap and may not be visible. Nevertheless, the sample sizes are as referenced throughout the paper (i.e., DCD = 10, TD = 11, TDM = 12).

To examine the effects of rhythmic auditory stimuli on tapping movements, statistical Group (3) × Task (2) mixed-design ANOVAs were carried out on the absolute period error and its standard deviation with Task (paced vs. unpaced) as the within-subjects factor. The analysis of the fast condition revealed main effects of Group [*F*_(2,30)_ = 6.68, *p* = 0.004, η_p_^2^ = 0.31] and Task for accuracy [*F*_(1,30)_ = 18.09, *p* < 0.001, η_p_^2^ = 0.38] and Group × Task interaction for variability [*F*_(2,30)_ = 5.67, *p* = 0.008, η_p_^2^ = 0.27]. Although children with DCD exhibited less accurate and more variable movements than their TDM peers, their motor accuracy and variability significantly improved when auditory cues were present [APE, *t*_(9)_ = 3.34, *p* = 0.008, *d* = 1.06; SD, *t*_(9)_ = 4.18, *p* = 0.002, *d* = 1.32] (see Figure 3). In the slow condition, we found a significant Group × Task interaction for accuracy [*F*_(2,30)_ = 5.53, *p* = 0.009, η_p_^2^ = 0.27] and main effects of Group [*F*_(2,30)_ = 8.01, *p* = 0.002, η_p_^2^ = 0.35] and Task [*F*_(1,30)_ = 11.53, *p* = 0.002, η_p_^2^ = 0.28] for variability measures. During slow unpaced tapping, children with DCD were significantly less accurate than their TD peers and more variable than the TDM group. Notably, the accuracy improved for all three groups when auditory cues were present [*t*_(9)_ = 5.65, *p* < 0.001, *d* = 1.79; TD, *t*_(10)_ = 3.85, *p* = 0.003, *d* = 1.16; TDM, *t*_(11)_ = 3.71, *p* = 0.003, *d* = 1.07], indicating that auditory rhythms may be even more beneficial during tasks that require greater inhibitory control, such as slow compared to fast tapping movements. Children with musical training seemed to benefit the most from the auditory rhythms as their error and variability significantly decreased (47.49 ± 4.27) compared to the DCD (95.07 ± 8.19) and TD groups (79.85 ± 7.73).

### EEG analyses

#### Power spectral density during tapping tasks

**Figure 4:**
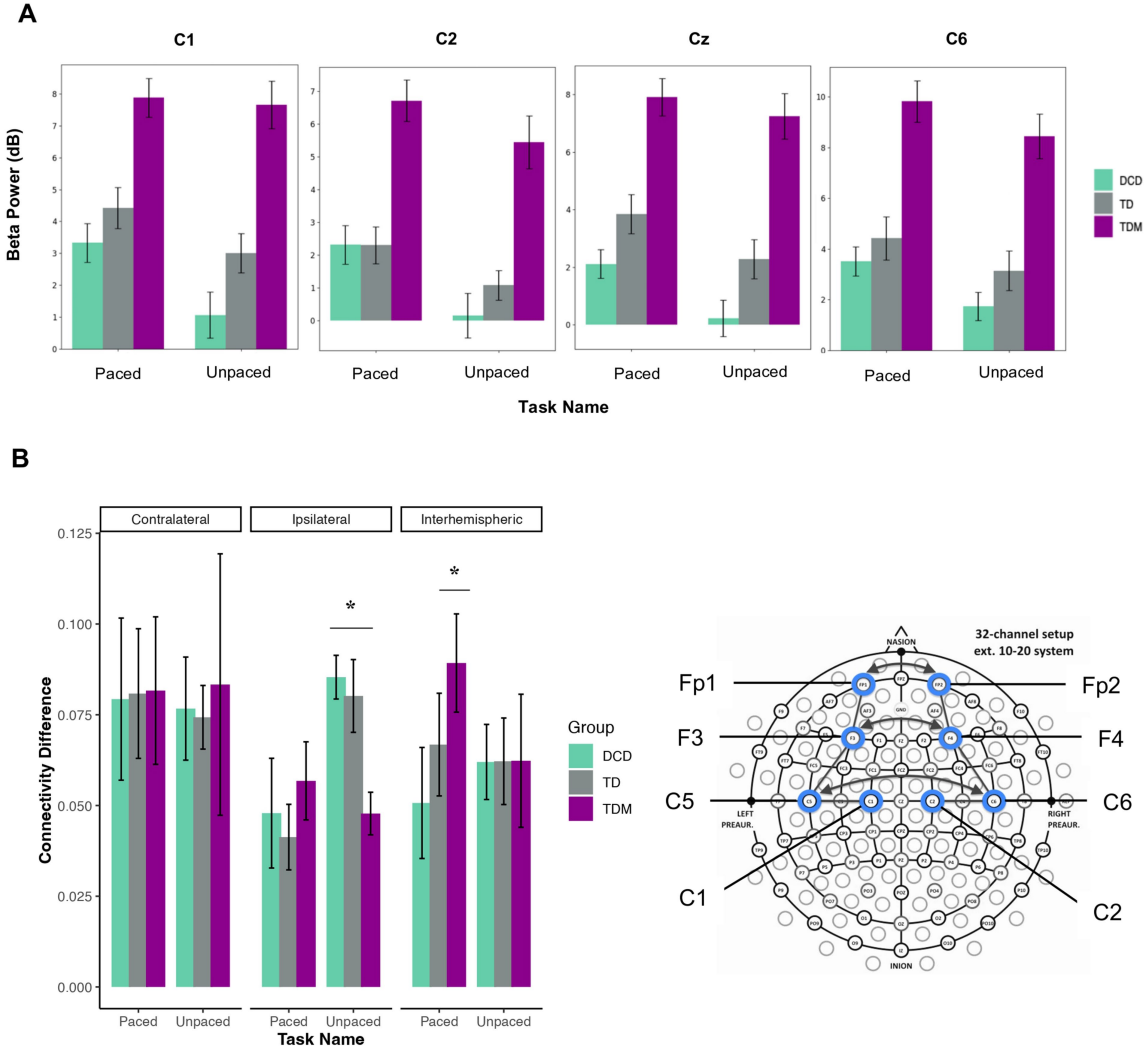
**(A)** Significant differences in the beta power were found for electrodes C1, C2, Cz, and C6 showing more power during paced than unpaced tapping in the slow condition (1,000 ms). Power spectral density (PSD) was calculated for each group and task. Error bars indicate the standard error of the mean. Units are in decibels (dB). **(B)** Functional connectivity changes in the beta band during paced and unpaced tapping at 1000 ms. Functional connectivity changes are reported for intrahemispheric contralateral (Fp1-F3, F3-C5, C5-C1), intrahemispheric ipsilateral (Fp2-F4, F4-C6, C6-C2), and interhemispheric groupings (Fp1-Fp2, F3-F4, C5-C6) in the slow tapping condition. Increased coherence was found within the ipsilateral network during unpaced tapping for the DCD and TD groups, while the musician group showed increased coherence in the interhemispheric network during paced tapping. Error bars indicate the standard error of the mean.

Statistical Group (3) × Task (2) mixed-design ANOVAs were carried out in the beta band for five electrodes across the sensorimotor network. Task-related changes in power spectral density were most evident during slow tapping. We found a main effect of Task for the C1 [*F*_(1,21)_ = 7.36, *p* = 0.013, η_p_^2^ = 0.26], Cz [*F*_(1,21)_ = 6.34, *p* = 0.020, η_p_^2^ = 0.23], C2 [*F*_(1,21)_ = 9.61, *p* = 0.005, η_p_^2^ = 0.31], and C6 electrodes [*F*_(1,21)_ = 5.95, *p* = 0.024, η_p_^2^ = 0.22]. DCD group displayed significantly lower neural beta power during unpaced compared to paced tapping tasks across C1 [*t*_(6)_ = 2.29, *p* = 0.031, *d* = 0.86], Cz [*t*_(6)_ = 2.32, *p* = 0.029, *d* = 0.88], and C2 electrodes [*t*_(6)_ = 2.72, *p* = 0.017, *d* = 1.03]. The effects for the C6 electrode were no longer significant after the Bonferroni correction. As illustrated in Figure 4A, the musician group consistently exhibited more beta power than other groups, although the groups did not significantly differ. No significant effects were found for the fast condition.

#### Changes in functional connectivity during tapping tasks

To examine changes in functional connectivity patterns between paced and unpaced tapping, statistical Group (3) × Task (2) × Connectivity (3) mixed-design ANOVAs were carried out with repeated measures on Task (paced vs. unpaced) and Connectivity (intrahemispheric contralateral, intrahemispheric ipsilateral, interhemispheric). As in the frequency domain, the analyses revealed no main or interaction effects for the fast condition. When auditory cues were present in the slow condition, functional connectivity in the intrahemispheric ipsilateral network decreased for the TD and DCD groups [Task × Connectivity interaction; *F*_(2, 42)_ = 5.58, *p* = 0.007, η_p_^2^ = 0.21; TD, *t*_(7)_ = 2.79, *p* = 0.013, *d* = 0.99; DCD, *t*_(6)_ = 2.19, *p* = 0.035, *d* = 0.83]. Only the results for the TD group persisted after the Bonferroni adjustment. In contrast, TD musicians displayed significantly increased coherence in the interhemispheric network during paced tapping [*t*_(8)_ = 2.82, *p* = 0.022, *d* = 0.94] and decreased coherence in the ipsilateral network during unpaced tapping compared to DCD and TD groups [DCD, *t*_(14)_ = 4.41, *p* < 0.001, *d* = 2.22; TD, *t*_(15)_ = 2.87, *p* = 0.011, *d* = 1.39] (see Figure 4B).

## Discussion

Neural and behavioral results are discussed in terms of (1) auditory-perceptual abilities and the role of musical training, (2) the effects of rhythmic auditory cues on motor performance, and (3) task-related changes in cortical networks.

### Auditory–perceptual abilities and the role of musical training

The current study focused on temporal aspects of auditory processing (i.e., duration and rhythm discrimination) rather than pitch or loudness perception. Our findings suggest that children with DCD have lower perceptual acuity for rhythmic timing than their TD peers with musical training. Although children with musical training also displayed decreased duration discrimination thresholds, group effects were no longer significant after the Bonferroni adjustment. While our findings should be further assessed with larger samples, the results are partially in contrast with earlier studies that reported decreased perceptual acuity on duration-based tasks (i.e., higher thresholds) in children with motor coordination difficulties compared to TD peers (Williams et al., 1992; Lundy-Ekman et al., 1991). A more recent study also found that duration deviations elicited delayed mismatch negativity (MMN) latencies in their sample of 6-7-year-old children at risk for DCD (Chang et al., 2021).

For rhythm-based timing, existing research offers limited and inconclusive results. For example, Chang and colleagues (Chang et al., 2021) found significantly decreased rhythm discrimination sensitivities and delayed P3a latencies in response to rhythm deviants in children at risk for DCD. In contrast, Roche and colleagues (Roche et al., 2016) found no group differences in rhythmic timing between children with DCD and their TD peers. While more research is critically needed, several factors may have contributed to these contrasting results, including differences in psychophysical procedures, inclusion criteria, and levels of musical training across samples. Two studies that reported duration timing difficulties (Williams et al., 1992; Lundy-Ekman et al., 1991) were published before the term DCD was introduced in 1994 (Blank et al., 2012); thus, it is possible that the “clumsy” children from their sample would not meet the current DSM-5 or ICD-10 criteria for DCD (American Psychiatric Association, 2013). Moreover, the lack of group differences in rhythm-based timing reported by Roche et al. (2016) should also be interpreted cautiously since their threshold estimation procedure was incongruous with commonly used adaptive psychophysical measurements (Treutwein, 1995). Most importantly, none of the existing studies controlled for the effects of musical training. Therefore, it is likely that their TD samples consisted of children with varying levels of musical expertise, resulting in heightened differences in perceptual thresholds.

Our findings underscore the positive effects of extensive musical training on rhythmic timing abilities and are congruous with the existing literature showing heightened sensitivity to rhythmic patterns in adult musicians (Chen et al., 2008; Herholz and Zatorre, 2012). Still, there is a continuous debate regarding the impact of individual pre-existing factors on enhanced musical abilities, such as individual cognitive abilities, musical aptitude, and demographic characteristics (Schellenberg, 2016). To control for these factors, we conducted standardized neuropsychological tests and parent questionnaires during the first study visit and found no significant differences in cognitive abilities and socioeconomic status between the groups. Our groups also did not significantly differ in duration thresholds, which suggests that TD musicians in our sample did not have pre-existing auditory acuity. Differences between duration and rhythm discrimination thresholds found in our study may be explained by neuroimaging literature, which indicates that duration and rhythm perception rely on relatively distinct neural circuitries wherein duration discrimination involves cerebellar-cortical pathways and the basal ganglia-cortical pathways support rhythm-based timing (Grube et al., 2010; Teki et al., 2011).

Here, we implemented a simple rhythm discrimination task involving an isochronous sequence of tones provided by the metronome (i.e., anisochrony detection with tones) to ensure the tasks were suitable for children. However, it is possible that more complex stimuli, such as auditory beats embedded in a musical excerpt (i.e., anisochrony detection with music), might be more sensitive in detecting rhythmic timing differences (Sowiński and Dalla, 2013). Indeed, a recent study employed a similar subset of sensorimotor tasks with children with ADHD and found vulnerabilities in rhythm discrimination with complex stimuli but not for simple rhythmic patterns (Puyjarinet et al., 2017). Differences in beat-based timing were also reported across several neurodevelopmental disorders that frequently co-occur with DCD (Lense et al., 1835). Overall, there is a gap in the literature concerning auditory-perceptual timing in DCD, and the existing studies provide conflicting findings and a limited understanding of the role of musical training.

### The effects of rhythmic auditory cues on motor performance

Findings from our tapping tasks are congruous with earlier studies showing that children with DCD are more variable and less accurate than their TD peers both when tapping to the auditory beat (Whitall et al., 2008; Whitall et al., 2006) and when maintaining a tapping pattern without external cues (Williams et al., 1992; Lundy-Ekman et al., 1991). The current study employed unimanual movements as they are largely unaffected by differences in cognitive abilities and cultural exposure (Whitall and Clark, 2018) and can be reliably studied in children. In the synchronization–continuation paradigm, children with DCD exhibited more variability than their peers, especially when coordinating movements at a slow pace. This is not surprising given the inter-tap interval of roughly 500 ms (2 Hz) is reported to be a preferred tapping rate in children (Repp, 2005; McAuley et al., 2006). However, recent literature points to additional factors that influence spontaneous motor tempo contributing to a variability of preferred rates (Desbernats et al., 2023). These factors may be both intrinsic (e.g., age, pathology, expertise) and extrinsic (type of task, physical training, external constraints). Developmental studies further indicate that children have a narrower frequency curve than adults, and their ability to entrain to slower rates broadens with age (Van Noorden and Moelants, 1999). For this reason, the slow condition imposed greater demands for all children in our sample.

When the auditory beat was present, TD musicians consistently displayed higher accuracy and lower variability, followed by TD children without musical training and children with DCD, indicating that long-term musical training can gradually influence how rhythmic periodicities are perceived and internalized, leading to enhanced auditory-motor coupling (Chen et al., 2008; Herholz and Zatorre, 2012). Likewise, it is plausible that children with musical training have developed enhanced beat alignment ability as extensive piano lessons require constant temporal adaptations across different tempi (Scheurich et al., 2020). Interestingly, this ability has been associated with formal musical training rather than current music playing (Spiech et al., 2023). In terms of clinical implications, our results indicate that children with DCD benefit from rhythmic auditory cues when coordinating movements. This finding is in line with existing literature showing positive effects of rhythmic entrainment in adults with movement disorders, such as Parkinson’s disease and stroke (Thaut and Abiru, 2010), and provides compelling evidence in favor of novel auditory/rhythm-based interventions for children with DCD.

### Task-related changes in cortical networks

To date, no studies have examined the neural dynamics underlying paced versus unpaced rhythmic movements in children with DCD and their TD peers. During paced tapping, the power spectral density in the beta band increased in the cortical motor network. Changes in power spectra closely mirrored the behavioral performance, with more beta power in musicians, followed by the TD and DCD groups. Although neurophysiological studies in DCD are lacking, research with adults showed a similar trend of increased beta power in musicians compared to individuals without musical training (Stegemöller et al., 2018) and decreased beta power in individuals with Parkinson’s disease (Stegemöller et al., 2016; Stegemöller et al., 2017).

We further examined functional connectivity patterns to gain insight into intrahemispheric and interhemispheric communication underlying rhythmic movements (Serrien and Brown, 2002) with high coherence values indicating an increased degree of synchronization (Fries, 2005). Our results showed that unpaced tapping involved a higher degree of neural communication in the ipsilateral intrahemispheric network, particularly in the DCD and TD groups (see Supplementary Table S4). Given their behavioral performance, this may indicate that unpaced tapping posed higher demands as children had to rely on their internal timing mechanisms to maintain the tapping rate. Indeed, improved motor performance has been associated with reduced recruitment of cortical and subcortical networks (Mayville et al., 2002; Pranjić et al., 2024). Studies in adults suggest that, while paced and unpaced performance engage overlapping neural networks, unpaced tapping requires additional processing resources in the medial circuitry (i.e., SMA) (Serrien, 2008; Lewis et al., 2004; Rao et al., 1997). In our study, augmented functional communication occurred in the ipsilateral fronto-central network, an area thought to be engaged with increased motor task complexity (Van Den Berg et al., 2011). Overall, these insights are congruent with developmental studies showing that cortico-cortical connectivity gradually decreases at the onset of locomotion due to synaptic pruning, contributing to the refinement of motor skills (Bell and Fox, 1996). Although age was not included as a covariate in our EEG analyses due to the lack of significant age differences between groups and in task performance, it is important to acknowledge that maturational differences in neural development may significantly influence EEG activity. For instance, prior work has shown that beta-band oscillatory activity develops across childhood and adolescence, with modulations in both spontaneous and task-related beta power reflecting the strengthening of top-down control mechanisms (Trevarrow et al., 2019). These beta dynamics follow distinct trajectories across the lifespan (Heinrichs-Graham et al., 2018). Moreover, developmental changes in functional connectivity also emerge with age and are thought to reflect the maturation of large-scale brain networks involved in executive function, motor planning, and inhibitory control (Smit et al., 2012; Uhlhaas et al., 2010). In younger children, these systems are less functionally integrated, which may result in lower beta coherence or atypical modulation patterns.

Interestingly, more interhemispheric connectivity was found in musicians when auditory cues were present. Similar findings were reported by Blais and colleagues (Blais et al., 2018), who found reduced interhemispheric fronto-central communication in teenagers with DCD who exhibited lower behavioral stability after motor practice. While future work involving larger samples is needed, neuroplastic changes in the corpus callosum have previously been reported in musicians (Schlaug et al., 1995; Vollmann et al., 2014), implying that musical training may enhance communication between hemispheres.

### Limitations and future directions

Several limitations should be considered when interpreting our findings. Consistent with common practice in DCD research, we used a cut-off point score of < 15^th^ percentile on the MABC-2 as our eligibility criterion for children with DCD. Nonetheless, future studies should investigate whether different percentile cut-offs (e.g., 5^th^ vs. 15^th^) can further characterize performance differences within the DCD group. Another limitation of the current study is the modest sample size, which constrains statistical power and may limit the generalizability of findings. This challenge is particularly relevant in EEG data, where high variability and individual differences can impact signal detection. While our multimodal, within-subject design mitigates some concerns, we acknowledge that the observed effects should be interpreted with caution until replicated in larger samples. Given the age of our participants, we employed simple auditory stimuli and unimanual tapping movements. However, more complex auditory-perceptual and motor tasks may offer greater sensitivity in detecting timing vulnerabilities. Taken together, future studies should involve larger samples and employ different levels of task complexity while controlling for the effects of musical training and the presence of co-occurring conditions (Lino and Chieffo, 2022; Pranjić et al., 2023).

## Conclusion

The results from this study provide novel insights into the behavioral and neural correlates of auditory-perceptual and auditory-motor timing in children with DCD and their TD peers with and without musical training. Our findings indicate that, despite vulnerabilities in rhythmic timing in both perceptual and motor tasks, children with DCD are less variable when coordinating movements with auditory rhythms. This suggests that children with DCD may benefit from auditory/rhythm-based interventions, as repeatedly demonstrated in adults with movement disorders. Given the strong association between rhythm perception and movement, future studies should continue to investigate the link between auditory and motor skills to identify the profiles of children who are most likely to benefit from such interventions. Characterizing potential subgroups of auditory-motor profiles is especially important considering the neurobiological heterogeneity in DCD.

## Data Availability

The data generated and/or analyzed during the current study are available from the corresponding author upon reasonable request.
