# Supplementary material for: Brain-behavior correlates of rhythmic timing and auditory-motor synchronization in children with developmental coordination disorder: an EEG study
Source: Front Hum Neurosci. 2025 Jun 5;19:1602580. doi: 10.3389/fnhum.2025.1602580 (PMC12176763; doi:10.3389/fnhum.2025.1602580)
Supplement: Supplementary file 1 [file Data_Sheet_1.pdf]

SUPPLEMENTARY MATERIAL

Brain-behavior correlates of rhythmic timing and auditory-motor synchronization in children with developmental coordination disorder: an EEG study

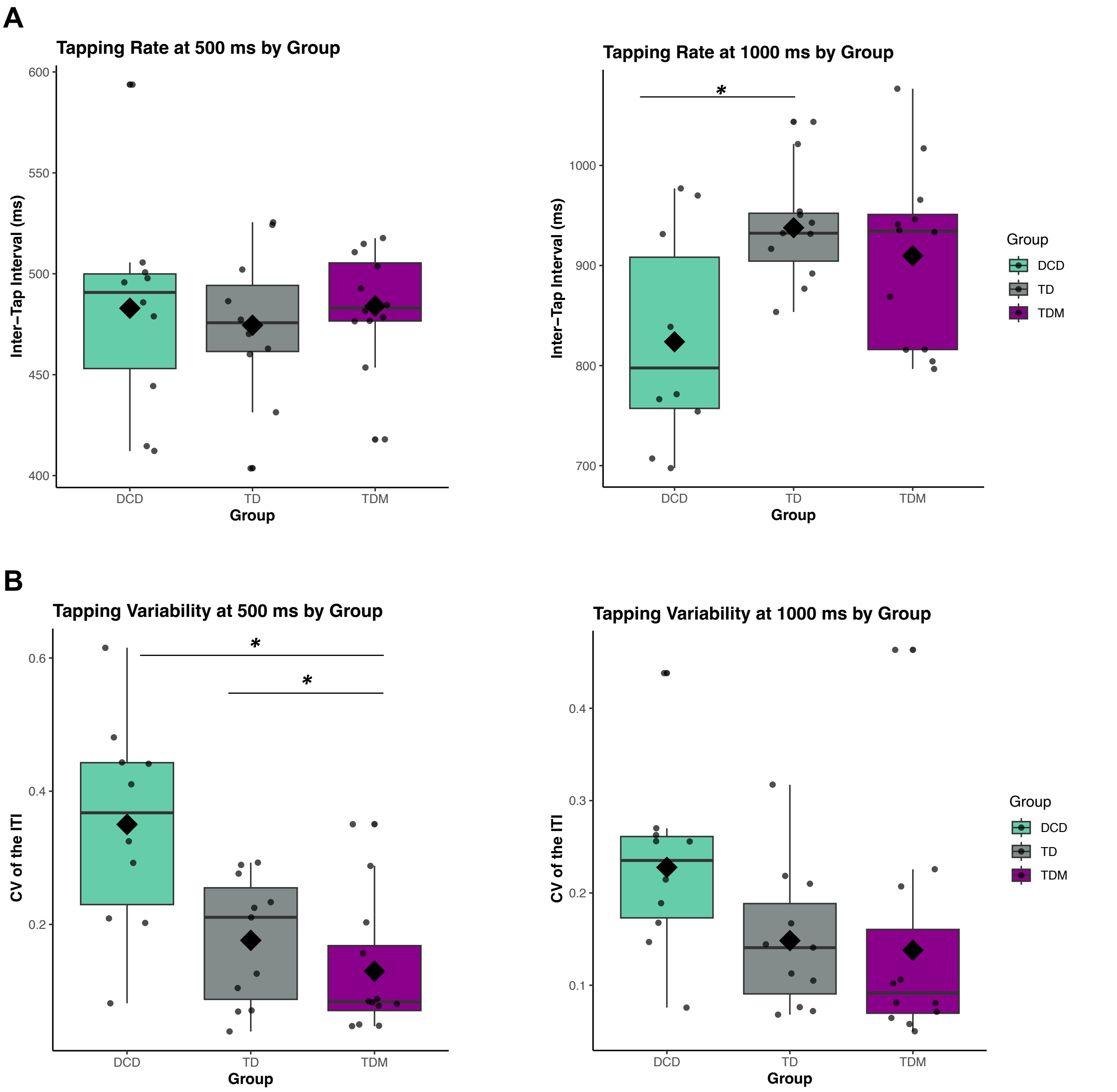

Figure S1. Unpaced performance in the fast (left) and slow (right) tapping conditions between groups. The synchronization-continuation paradigm was used to assess the internal timing mechanisms in children. (A) The mean inter-tap interval (ITI) indicates tapping consistency in maintaining the internal representation of the beat. Values closer to 500 ms (left) and 1000 ms (right) represent increased consistency. (B) The coefficient of variation of the ITI reflects motor variability. Lower values indicate less variability (i.e., better performance). *Denotes significance at $p_{adj} < .016$.

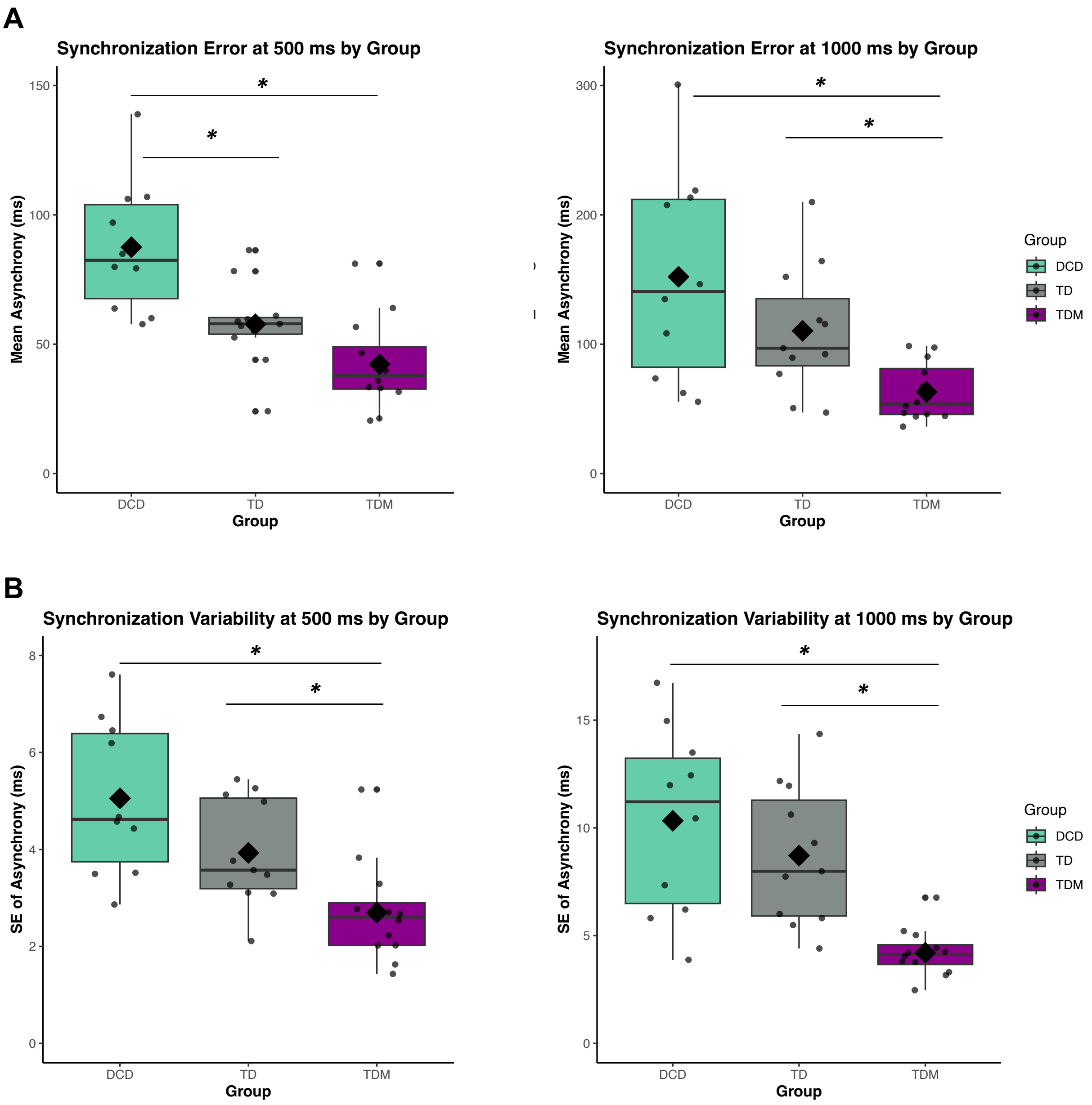

Figure S2. Paced performance in the fast (left) and slow (right) tapping conditions between groups. The synchronization paradigm was used to assess the participants' ability to align their taps with the auditory beat. (A) The mean phase synchronization error reflects how accurately the participants coordinated the movement to an auditory rhythm. Lower error values indicate better synchronization. (B) Synchronization variability is reported as the standard error (SE), wherein lower values indicate better performance. *Denotes significance at $p_{adj} < .016$.
